# Net water uptake, a neuroimaging marker of early brain edema, as a predictor of symptomatic intracranial hemorrhage after acute ischemic stroke

**DOI:** 10.3389/fneur.2022.903263

**Published:** 2022-07-27

**Authors:** Tianqi Xu, Jianhong Yang, Qing Han, Yuefei Wu, Xiang Gao, Yao Xu, Yi Huang, Aiju Wang, Mark W. Parsons, Longting Lin

**Affiliations:** ^1^Department of Neurology, Ningbo First Hospital, Ningbo Hospital of Zhejiang University, Ningbo, China; ^2^Department of Neurosurgery, Ningbo First Hospital, Ningbo Hospital of Zhejiang University, Ningbo, China; ^3^Key Laboratory of Precision Medicine for Atherosclerotic Diseases of Zhejiang Province, Ningbo, China; ^4^Department of Neurology, Ningbo Fourth Hospital, Ningbo, China; ^5^Sydney Brain Center, University of New South Wales, Sydney, NSW, Australia; ^6^Department of Neurology, Liverpool Hospital, Sydney, NSW, Australia

**Keywords:** brain edema, multimodal CT, net water uptake, symptomatic intracranial hemorrhage (sICH), acute ischemic stroke (AIS), mechanical thrombectomy (MT)

## Abstract

**Objective:**

We hypothesized that quantitative net water uptake (NWU), a novel neuroimaging marker of early brain edema, can predict symptomatic intracranial hemorrhage (sICH) after acute ischemic stroke (AIS).

**Methods:**

We enrolled patients with AIS who completed admission multimodal computed tomography (CT) within 24 h after stroke onset. NWU within the ischemic core and penumbra was calculated based on admission CT, namely NWU-core and NWU-penumbra. sICH was defined as the presence of ICH in the infarct area within 7 days after stroke onset, accompanied by clinical deterioration. The predictive value of NWU-core and NWU-penumbra on sICH was evaluated by logistic regression analyses and the receiver operating characteristic (ROC) curve. A pure neuroimaging prediction model was built considering imaging markers, which has the potential to be automatically quantified with an artificial algorithm on image workstation.

**Results:**

154 patients were included, of which 93 underwent mechanical thrombectomy (MT). The median time from symptom onset to admission CT was 262 min (interquartile range, 198–368). In patients with MT, NWU-penumbra (OR =1.442; 95% CI = 1.177–1.766; *P* < 0.001) and NWU-core (OR = 1.155; 95% CI = 1.027–1.299; *P* = 0.016) were independently associated with sICH with adjustments for age, sex, time from symptom onset to CT, hypertension, lesion volume, and admission National Institutes of Health Stroke Scale (NIHSS) score. ROC curve showed that NWU-penumbra had better predictive performance than NWU-core on sICH [area under the curve (AUC): 0.773 vs. 0.673]. The diagnostic efficiency of the predictive model was improved with the containing of NWU-penumbra (AUC: 0.853 vs. 0.760). A pure imaging model also presented stable predictive power (AUC = 0.812). In patients without MT, however, only admission NIHSS score (OR = 1.440; 95% CI = 1.055–1.965; *P* = 0.022) showed significance in predicting sICH in multivariate analyses.

**Conclusions:**

NWU-penumbra may have better predictive performance than NWU-core on sICH after MT. A pure imaging model showed potential value to automatically screen patients with sICH risk by image recognition, which may optimize treatment strategy.

## Introduction

Brain edema is an important pathophysiological process after acute ischemic stroke (AIS). The pathophysiological mechanisms are primarily cytotoxic edema, angiogenic edema, and cerebrospinal fluid influx (1, 2). Progressive edema will lead to irreversible tissue injury, secondary neurological deterioration, and poor outcome (3). Current assessments of brain edema degree are mainly based on follow-up non-contrast computed tomography (NCCT), as brain edema peaks around 3 days after stroke onset (4). Imaging characteristics such as shrunken sulci, low density around infarct core, midline shift, and even brain hernia suggest brain edema indirectly (5). However, there are few neuroimaging markers able to detect brain edema early, directly, and quantitatively, and none of these neuroimaging markers were available in CT until recently (6).

Quantitative net water uptake (NWU) is a relatively novel neuroimaging marker of early brain edema after AIS, first described in 2016 based on admission multimodal CT (7). Researchers subsequently reported that NWU within the ischemic core can identify patients within the thrombolysis time window and predict poor outcomes at 90 days, and those with malignant edema (8–10). In the hyperacute stage of AIS, cytotoxic edema will lead to endothelial cell swelling, further blood-brain barrier (BBB) breakdown, increased permeability, and ultimately may contribute to hemorrhagic transformation (HT). Previous research reported that NWU within the ischemic core was a predictor of HT in patients with successful reperfusion (11). In this study, NWU within the ischemic core and the ischemic penumbra were calculated separately. The predictive values of NWU-core and NWU-penumbra on symptomatic intracranial hemorrhages (sICH) after AIS were further investigated.

## Methods

### Patients

This is a single-institute retrospective study. The clinical and imaging data were obtained from an established database, which contains data from all patients with AIS admitted to the Emergency Department at Ningbo First Hospital (Advanced Stroke Center) between July 2017 and September 2019. Inclusion criteria: (1) acute anterior circulation infarction; (2) admission multimodal CT including NCCT, CT angiography (CTA), and CT perfusion (CTP) were completed within 24 h after symptom onset. In patients with wake-up stroke, we estimated the time of stroke onset by taking the midpoint between sleep time and wake time (12); (3) follow-up CT scans performed at 24 h and 1 week after stroke onset or immediately in case of clinical deterioration; (4) the absence of intracranial hemorrhage, brain tumor, or preexisting infarctions in admission NCCT. The thrombolysis in cerebral infarction (TICI) grading scale is used to define endpoints of successful revascularization (13).

### Definition of sICH

According to the European Cooperative Acute Stroke Study (ECASS) II criteria, hemorrhagic transformation (HT) of infarcted brain tissue can be divided into four types, including HI1 (scattered small petechiae, no mass effect), HI2 (confluent petechiae, no mass effect), PH1 (hematoma within infarcted tissue, occupying <30%, no substantive mass effect), and PH2 (hematoma occupying 30% or more of the infarcted tissue, with obvious mass effect). sICH was defined as the presence of hemorrhagic hyperdensity in the infarction area on follow-up CT scans within 7 days, accompanied by clinical deterioration (an NIHSS score increase ≥ 4 from baseline) (14, 15) with no explanation for the clinical deterioration other than secondary intracerebral hemorrhage.

### Imaging analysis

**Figure 1:**
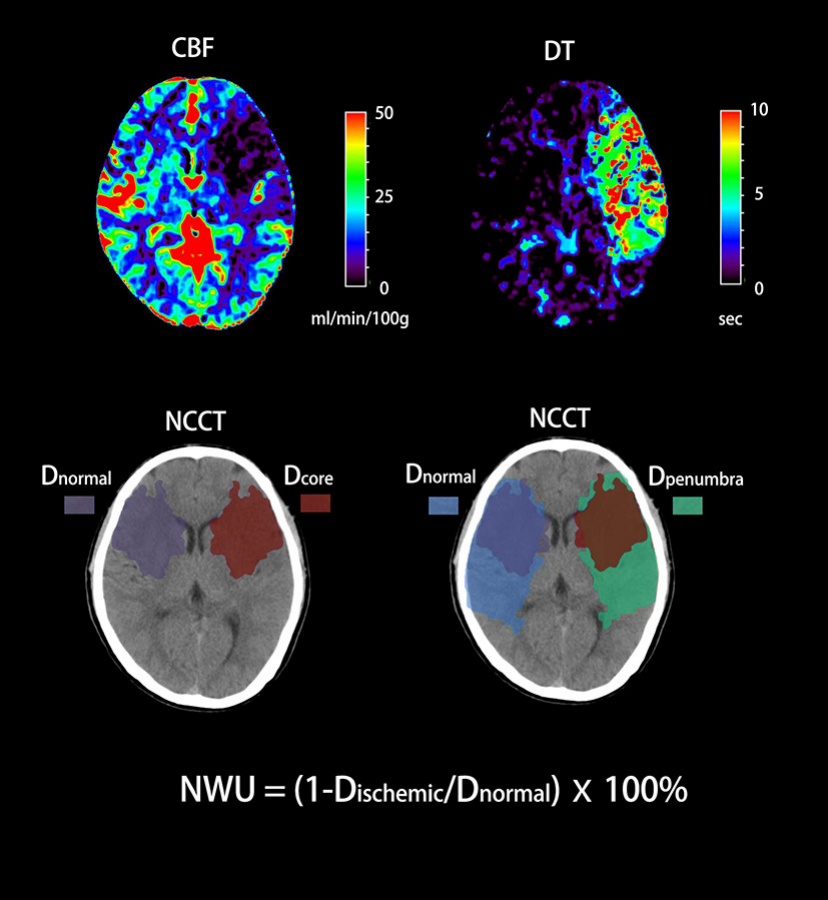
Quantification of NWU-core and NWU-penumbra in admission CT. CBV and DT maps based on CTP were used to identify the ischemic core and penumbra with the setting of different thresholds. The mean density of the ischemic core, ischemic penumbra, and normal tissue derived from mirrored contralateral were then calculated (*D*_r*e*_, *D*_p*enra*_, and *D*_n*ra*_).

Admission multimodal CT was conducted by a 320-slice scanner (Toshiba Aquilion ONE, Canon Medical Imaging, Tokyo, Japan) with 320 axial sections and 0.5 mm thickness. All imaging data were processed by commercial software (MIstar, Apollo Medical Imaging Technology, Melbourne, VIC, Australia). CTP was used to identify the ischemic core and ischemic penumbra. The threshold of ischemic core was delay time (DT) > 3 s and cerebral blood flow (CBF) <30%. The threshold of ischemic penumbra was DT > 3 s and CBF > 30% (16). Regions of interest (ROIs) corresponding to the ischemic core and penumbra according to these thresholds were drawn manually, and mirrored contralateral ROIs were generated automatically and defined as normal tissue. These ROIs were then superimposed on NCCT to measure CT density (*D*_r*e*_, *D*_p*enra*_, and *D*_n*ra*_) with Hounsfield units (HU) between 20 and 80 (Figure 1). NWU-core was defined as an elevated volume of water (Δ _a*ter*_) per unit volume of ischemic core (_core_). Similarly, NWU-penumbra was defined as Δ _a*ter*_ per unit volume of ischemic penumbra (_penumbra_), according to the following equations (6, 7):


$$NWU_{core}=\frac{V_{water}}{V_{core}}=\left(1-\frac{D_{core}}{D_{normal}}\right)\times 100\%\;$$

$$NWU_{penumbra}=\frac{V_{water}}{V_{penumbra}}=\left(1-\frac{D_{penumbra}}{D_{normal}}\right)\times 100\%\;$$

### Statistical methods

We performed the statistical analyses under the supervision of professional statisticians. SPSS version 22.0 (SPSS Inc, Chicago, IL, USA), MedCalc version 20.027 (MedCalc Software Ltd, Ostend, Belgium), and GraphPad Prism version 8.0 (GraphPad Software, San Diego, CA, USA) were used in these analyses. Baseline characteristics were compared between patients with and without sICH. For continuous variables that followed a normal distribution, data were expressed as mean ± SD, and the *t*-test was used for group comparison. For non-normal continuous variables, data were presented in the form of the medians (interquartile ranges), and the Mann–Whitney *U* test was applied. Categorical variables were expressed as frequencies (%) using the χ^2^ test or *Fisher's* test as appropriate. Univariate logistic regression analyses were used to explore the associations between sICH, NWU-core, and NWU-penumbra. Backward stepwise multivariate logistic regression analyses were then performed. We used age, sex, admission NIHSS score, time from symptom onset to CT, lesion volume, and variables with *P* < 0.10 in the univariate analyses as covariates. The variance inflation factor (VIF) was calculated to examine multicollinearity among variables included in regression models. Predictive models containing NWU-core and NWU-penumbra were further built. The predictive power of NWU-core, NWU-penumbra, and regression models were measured by receiver operating characteristic (ROC) curve analysis.

## Results

### Baseline characteristics of enrolled patients

A total of 154 patients with acute anterior circulation ischemic stroke were included in the study. There were 58 cases of HT (37.7%), including 8 cases of HI1 (5.2%), 24 cases of HI2 (15.6%), 12 cases of PH1 (7.8%), and 14 cases of PH2 (9.1%). sICH occurred in 28 patients (18.2%). Table 1 shows the baseline characteristics of patients. The median time from symptom onset to multimodal CT was 262 min (interquartile range, 198–368). Compared with non-sICH patients, patients with sICH have higher median admission NIHSS score [21 (15–28) vs. 17 (12–21), *P* = 0.008], higher median ischemic core volume [48 (32–62) vs. 24 (9–45), *P* < 0.001], higher median NWU-core [8.2 (5.8–10.9) vs. 5.3 (2.7–7.9), *P* = 0.015] and higher median NWU-penumbra [3.6 (2.1–5.1) vs. 0.9 (−1.5 to 3.5), *P* < 0.001].

**Table 1:** Baseline characteristics of enrolled patients.

	**Non-sICH (*****n*** = **126)**	sICH^*^(***n*** = **28)**	***P*** **-value**
**Demographics**
Age, mean ± SD	69 ± 13	70 ± 11	0.353
Sex, male (%)	74 (58.7)	13 (46.4)	0.293
**Comorbidities**
Hypertension, *n* (%)	73 (57.9)	22 (78.6)	**0.053**
Diabetes mellitus, *n* (%)	11 (8.7)	6 (21.4)	**0.088**
Atrial fibrillation, *n* (%)	75 (59.5)	19 (67.9)	0.522
Coronary heart disease, *n* (%)	15 (12.0)	4 (14.3)	0.753
**Stroke etiology**			0.711
Atherothrombotic, *n* (%)	27 (21.4)	4 (14.3)	
Cardioembolic, *n* (%)	72 (57.1)	18 (64.3)	
Others, *n* (%)	27 (21.4)	6 (21.4)	
NIHSS score, median (IQR)	17 (12–21)	21 (15–28)	**0.008**
Time from symptom onset to CT, min, median (IQR)	253 (194–366)	310 (242–398)	0.100
**Imaging biomarkers**
Ischemic core volume, ml, median (IQR)	24 (9–45)	48 (32–62)	**<0.001**
Ischemic penumbra volume, ml, median (IQR)	66 (33–102)	87 (57–136)	**0.060**
NWU-core, %, median (IQR)	5.3 (2.7–8.9)	8.2 (5.8–10.9)	**0.015**
NWU-penumbra, %, median (IQR)	0.9 (−1.5 to 3.5)	3.6 (2.1–5.1)	**<0.001**
**Treatment**			**0.069**
Mechanical thrombectomy, *n* (%)	51 (40.5)	18 (64.3)	
Intravenous thrombolysis, *n* (%)	44 (34.9)	4 (14.1)	
Bridging therapy, *n* (%)	19 (15.1)	5 (17.9)	
Antiplatelet agents, *n* (%)	12 (9.5)	1 (3.6)	

*sICH, symptomatic intracranial hemorrhage; NIHSS, National Institutes of Health Stroke Scale; NWU-core, net water uptake within ischemic core; NWU-penumbra, net water uptake within ischemic penumbra*.

*For continuous variables that follow a normal distribution, data were expressed as mean ± SD, the t-test was used for group comparison. For non-normal continuous variables, data were presented in the form of the medians (interquartile ranges), and the Mann-Whitney U-test was applied. Categorical variables were expressed as frequencies (percentages), using the χ^2^ test or Fisher's test as appropriate*.

* *sICH was defined in reference to ECASSii criterion as hemorrhagic hyperintense in the infarction area scanned by CT within 1 week from symptom onset, accompanied by an increase of at least 4 points in the NIHSS score from baseline. The bold values means p <0.1*.

### Univariate logistic regression analysis for risks of sICH in patients stratified by reperfusion therapies

**Figure 2:**
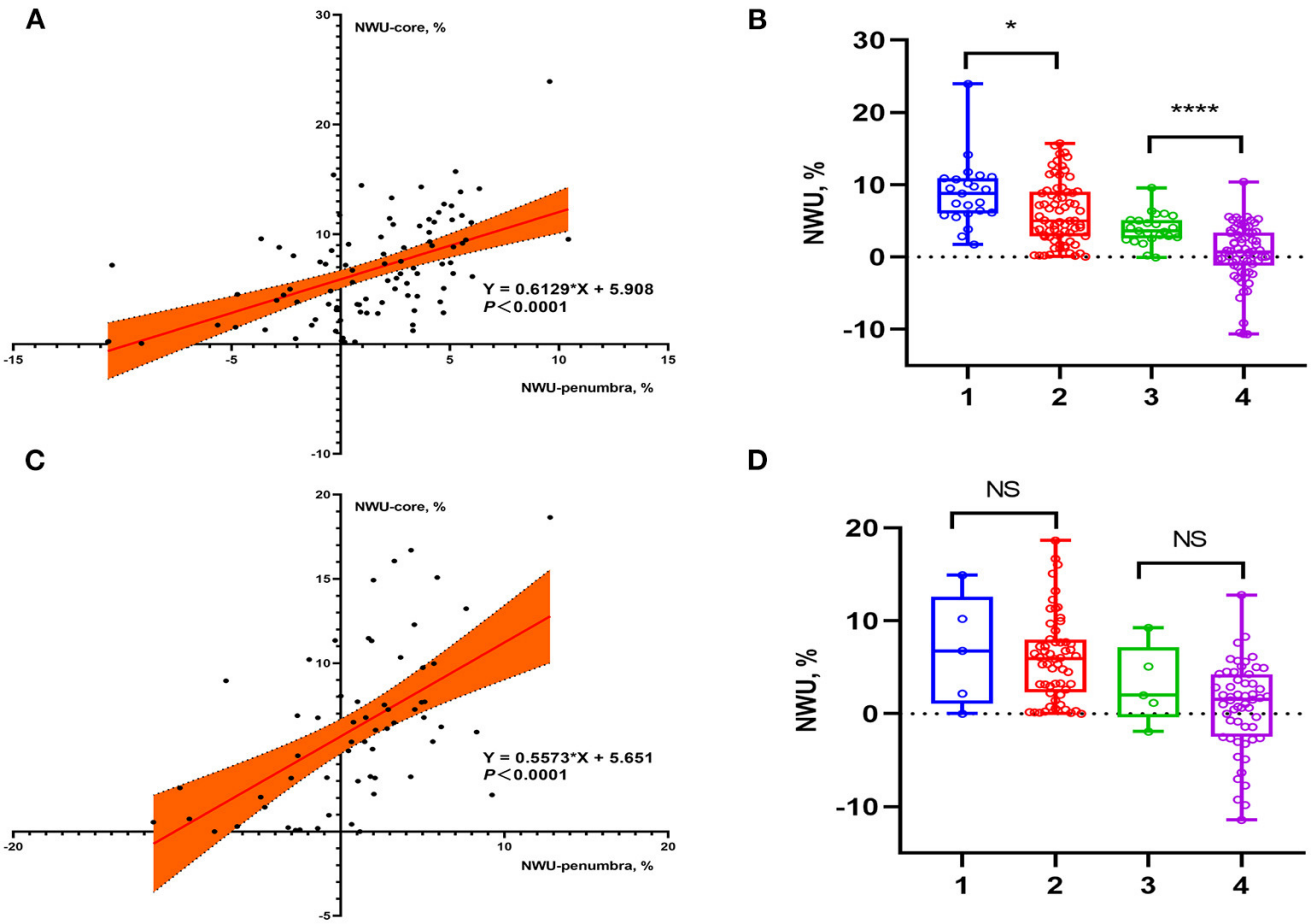
Data distributions of NWU-core and NWU-penumbra in patients stratified by reperfusion therapies. **(A)** Scatter plot showed the relationship between NWU-core and NWU-penumbra in patients with MT. **(B)** Group 1: NWU-core of sICH patients, group 2: NWU-core of non-sICH patients, group 3: NWU-penumbra of sICH patients, group 4: NWU-penumbra of non-sICH patients. Box plot showed that in patients with MT, sICH group had higher NWU-core [8.8 (6.0–10.9) vs. 5.0 (2.8–9.0), *P* = 0.013] and NWU-penumbra [2.6 (3.6–5.1) vs. 0.7 (−1.2 to 3.4), *P* < 0.001] than those without sICH. **(C)** Scatter plot showed the relationship between NWU-core and NWU-penumbra in patients without MT. **(D)** Group 1: NWU-core of sICH patients, group 2: NWU-core of non-sICH patients, group 3: NWU-penumbra of sICH patients, group 4: NWU-penumbra of non-sICH patients. Box plot showed that in patients without MT, there was no statistical difference in NWU-core [6.8 (1.1–12.6) vs. 6.0 (2.3–8.0), *P* = 0.885] and NWU-penumbra [2.0 (−0.4 to 7.2) vs. 1.5 (−2.8 to 4.1), *P* = 0.318] between sICH and non-sICH group. * *p* < 0.05; ** *p* < 0.01; *** *p* < 0.001; **** *p* < 0.0001.

Among the 154 enrolled patients, 93 underwent MT, of which 73 patients (78.5%) received successful reperfusion according to the TICI grading scale. Of the 61 patients who did not receive MT, 48 underwent intravenous thrombolysis and 13 received antiplatelet agents only. Supplementary Table 1 shows patient's demographics of group MT vs. non-MT and indicates that 23 (82.1%) of the 28 patients with sICH underwent MT. Figure 2 shows the data distributions of NWU-core and NWU-penumbra. In the MT group, patients with sICH had higher NWU-core [8.8 (6.0–10.9) vs. 5.0 (2.8–9.0), *P* = 0.013] and higher NWU-penumbra [2.6 (3.6–5.1) vs. 0.7 (−1.2 to 3.4), *P* < 0.001]. Univariate analysis showed that hypertension (OR = 3.812; 95% CI = 1.274–11.406; *P* = 0.017), ischemic core volume (OR = 1.014; 95% CI = 1.001–1.027; *P* = 0.034), NWU-core (OR = 1.143; 95% CI = 1.024–1.277; *P* = 0.018), and NWU-penumbra (OR =1.431; 95% CI = 1.159–1.766; *P* = 0.001) were associated with sICH after MT. However, in patients without MT, none of the imaging biomarkers showed significance, only admission NIHSS score (OR = 1.237; 95% CI = 1.066–1.434; *P* = 0.005) and diabetes mellitus (OR = 11.778; 95% CI = 1.394–99.511; *P* = 0.024) were significantly correlated with sICH (Table 2). The associations between NWU-core and NWU-penumbra with different types of HT are shown in Supplementary Table 2.

**Table 2:** Univariate logistic regression analysis for risk of sICH in patients stratified by reperfusion therapies.

	**With MT (*****n*** = **93)**		**Without MT (*****n*** = **61)**	
	**OR (95% CI)**	***P*** **-value**	**OR (95% CI)**	***P*** **-value**
**Clinical characteristics**
Age	1.020 (0.981–1.060)	0.322	0.996 (0.919–1.079)	0.926
Male	0.818 (0.318–2.100)	0.676	0.342 (0.053–2.227)	0.262
Hypertension	3.812 (1.274–11.406)	**0.017**	1.744 (0.181–16.778)	0.630
Diabetes mellitus	1.042 (0.388–2.797)	0.935	11.778 (1.394–99.511)	**0.024**
Atrial fibrillation	1.632 (0.442–6.021)	0.462	3.467 (0.364–33.001)	0.280
Coronary heart disease	1.605 (0.435–5.926)	0.478	NA	0.999
Stroke etiology	1.018 (0.634–1.636)	0.940	1.221 (0.523–2.850)	0.644
NIHSS score	1.054 (0.980–1.134)	0.157	1.237 (1.066–1.434)	**0.005**
Time from symptom onset to CT	1.001 (0.998–1.003)	0.657	1.003 (0.996–1.009)	0.412
**Imaging biomarkers**
Ischemic core volume	1.014 (1.001–1.027)	**0.034**	1.009 (0.996–1.022)	0.194
Ischemic penumbra volume	1.003 (0.994–1.011)	0.542	0.995 (0.974–1.016)	0.617
NWU-core	1.143 (1.024–1.277)	**0.018**	1.035 (0.858–1.250)	0.717
NWU-penumbra	1.431 (1.159–1.766)	**0.001**	1.131 (0.911–1.405)	0.265

*sICH, symptomatic intracranial hemorrhage; MT, mechanical thrombectomy; NIHSS, National Institutes of Health Stroke Scale; NWU-core, net water uptake within ischemic core; NWU-penumbra, net water uptake within ischemic penumbra. The bold values means p <0.1*.

### Multivariate logistic regression analysis for predictive models of sICH in patients stratified by reperfusion therapies

Table 3 shows four different predictive models of sICH in patients with MT. Model 1 was built without NWU for subsequent comparison. Models 2 and 3 included NWU-core and NWU-penumbra separately as they were correlated (Spearman correlation coefficient = 0.555; *P* < 0.001). Model 4 contained only imaging markers. We found that NWU-penumbra (OR = 1.442; 95% CI = 1.177–1.766; *P* < 0.001) and NWU-core (OR = 1.155; 95% CI = 1.027–1.299; *P* = 0.016) were independently associated with sICH after adjustment for age, sex, admission NIHSS score, time from symptom onset to CT, lesion volume, and hypertension. The imaging-only model contained ischemic core volume (OR = 1.017; 95% CI = 1.001–1.033; *P* = 0.034) and NWU-penumbra (OR = 1.412; 95% CI = 1.150–1.735; *P* = 0.001). In patients without MT, only admission NIHSS score (OR = 1.440; 95% CI = 1.055–1.965; *P* = 0.022) was independently associated with sICH.

**Table 3:** Multivariable logistic regression analysis for predictive models of sICH in patients with MT.

	**OR (95% CI)**	***P*** **-value**
**Model 1: without NWU**
Hypertension	3.663 (1.199–11.186)	**0.023**
Ischemic core volume	1.013 (0.999–1.026)	0.067
**Model 2: containing NWU-core**
NWU-core	1.155 (1.027–1.299)	**0.016**
Hypertension	4.338 (1.330–14.150)	**0.015**
Ischemic core volume	1.012 (0.998–1.026)	0.091
**Model 3: containing NWU-penumbra**
NWU-penumbra	1.442 (1.177–1.766)	**<0.001**
Hypertension	5.209 (1.466–18.508)	**0.011**
Ischemic core volume	1.017 (1.000–1.034)	0.052
**Model 4: pure imaging biomarkers**
NWU-penumbra	1.412 (1.150–1.735)	**0.001**
Ischemic core volume	1.017 (1.001–1.033)	**0.034**

*sICH, symptomatic intracranial hemorrhage; MT, mechanical thrombectomy; NWU-core, net water uptake within ischemic core; NWU-penumbra, net water uptake within ischemic penumbra*.

*Model 1 was built without NWU for subsequent comparison. Model 2 containing NWU-core was adjusted by age, sex, baseline NIHSS score, time from symptom onset to CT. Model 3 containing NWU-penumbra was adjusted by age, sex, baseline NIHSS score, time from symptom onset to CT. Model 4 was a pure imaging predictive model without the adjustment of clinical characteristics. The bold values means p <0.1*.

### ROC curve analyses of sICH in patients with MT

**Figure 3:**
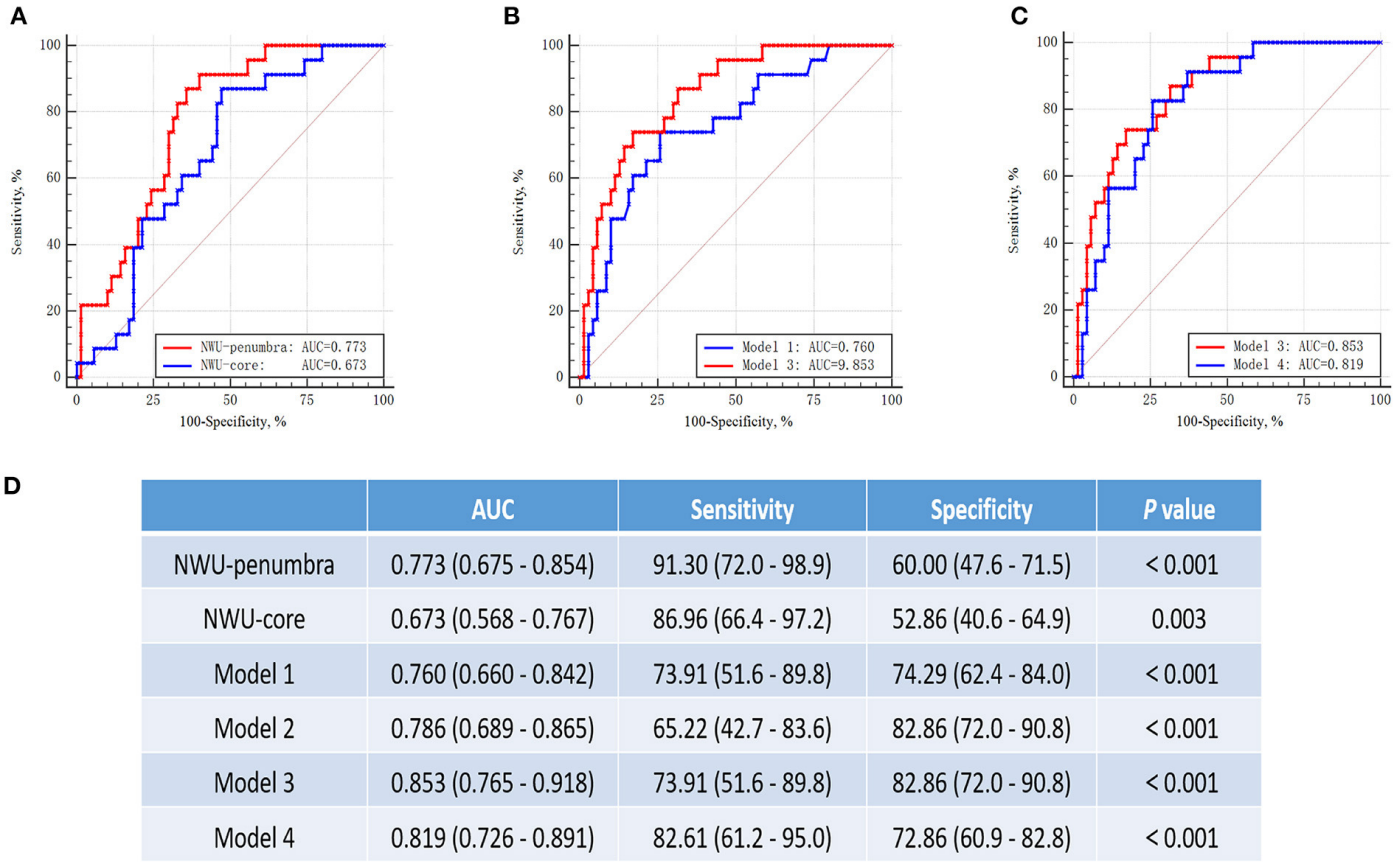
ROC curves for prediction of sICH in patients with mechanical thrombectomy. **(A)** The ROC curves of NWU-penumbra vs. NWU-core (AUC: 0.773 vs. 0.673). **(B)** The ROC curves of model 1 vs. model 3 (AUC: 0.760 vs. 0.853). **(C)** The curves of model 3 vs. model 4 (AUC: 0.853 vs. 0.812). **(D)** Table showed the data of ROC curve analysis.

With multivariate regression analysis, we discovered that NWU-penumbra and NWU-core were independently associated with sICH in the MT group and built four different predictive models. ROC curve analyses were conducted to test the predictive power of individual variables for models predicting sICH. Figure 3 shows that the area under the curve (AUC) of NWU-penumbra was higher than that of NWU-core (0.773 vs. 0.673). The diagnostic efficiency of the predictive model was improved with the inclusion of NWU-penumbra (AUC of model 3 vs. model 1 = 0.853 vs. 0.760). The pure imaging predictive model also demonstrated a stable predictive effect (AUC of model 4 = 0.812). It is also noteworthy that NWU-penumbra demonstrated high sensitivity (91.30; 95% CI = 72.0–98.9) in predicting sICH with an optimal cut point of 1.7%. Supplementary Figure 1 shows the illustrative cases for high NWU with sICH vs. low NWU without sICH.

## Discussion

In this research, quantitative NWU was used to assess the degree of early post-stroke brain edema within the ischemic core and penumbra separately. We found that NWU-penumbra may have a better value than NWU-core for predicting sICH after MT, and a pure imaging model containing NWU-penumbra and ischemic core volume showed a potential value for predicting sICH after MT independent of clinical variables.

Quantitative NWU, based on admission multimodal CT, has been described as a novel neuroimaging marker of early brain edema (6, 7). The NWU began immediately after AIS with the extravasation of fluid into ischemic tissue through the BBB, which may allow the detection of ionic edema earlier and more sensitively than previous methods (17). Previous studies reported that NWU within the ischemic core was an independent predictor for patients with AIS developing malignant edema (9, 18, 19) and poor neurological outcomes (8, 10). The cerebral ischemic lesion consists of a central irreversible necrotic zone and reversible peripheral ischemic penumbra. One of the main findings in our study was that NWU-penumbra demonstrates better predictive performance than NWU-core on sICH after MT. The most likely mechanisms for this water uptake involve disruption of the BBB caused by cytotoxic edema and angiogenic edema (20, 21). Although speculative, the presence and elevation of early brain edema within the penumbra may indicate a more serious tissue injury and damage expansion. Timely reperfusion can salvage ischemic penumbra but may also aggravate BBB destruction due to reperfusion injury. Tissue edema may compress the small blood vessels, leading to the impairment of microcirculation, which may contribute to vascular endothelial injury and secondary hemorrhage (22). Previous studies indicated that NWU-core was associated with HT within 36 h after successful endovascular treatment (11, 23). In this study, we focused on sICH within 7 days after stroke onset, which was defined as the presence of HT accompanied by clinical deterioration (the NIHSS score increased by more than 4 points from baseline). From a clinical perspective, sICH is strongly related to patient outcomes and often leads to longer hospital stays and more attention from neurologists to optimize treatment strategies, such as reducing intraoperative heparin dosage, avoiding dual antiplatelet, and appropriate blood pressure management. This study showed that in patients with AIS without MT, the predictive value of NWU on sICH might be overwhelmed by the admission NIHSS score. This result may be explained by an insufficient number of patients and a comparably low incidence of sICH among patients without MT. Patients who received MT tended to have larger vessel occlusions and had a higher recanalization probability, which may result in more severe reperfusion injury and BBB disruption. The surgery itself can also cause damage to blood vessels.

In this study, we examined several different models for predicting sICH after MT and found that the inclusion of NWU-penumbra significantly improved the predictive power of such models. Importantly, the pure imaging model containing NWU-penumbra and ischemic core volume also showed a stable predictive value for sICH after MT. In most hospitals, CT at admission is the standard care for patients with AIS, with the advantages of being widely and rapidly available and intrinsically quantitative (24). Therefore, compared with other predictors for sICH, such as serum occludin level, interleukin-33, and platelet-to-lymphocyte ratio (25–27), imaging makers would be comparatively simple and practical. A pure imaging model that does not include clinical characteristics may have the potential to be calculated automatically on the image workstation through deep neural networks (28). In future studies, the imaging model may be served as part of the computer-aided diagnosis systems to screen patients at sICH risk before treatment.

Our study has some limitations. Foremost, is the fact that it was a retrospective and single-center study. Further external validation trials in larger cohorts are required before clinical implementation. Secondly, the incidence of sICH was relatively low in the non-MT group, which may result in insufficient statistical power.

In conclusion, we found that NWU-penumbra had better predictive performance than NWU-core on sICH after MT. The pure imaging model based on admission CT may automatically flag patients with elevated sICH risk. It helps further hypothesis generation to better identify patients at risk of reperfusion injury or parenchymal hemorrhagic transformation in patients undergoing endovascular thrombectomy.

## Data availability statement

The raw data supporting the conclusions of this article will be made available by the authors, without undue reservation.

## Ethics statement

The studies involving human participants were reviewed and approved by Ningbo First Hospital, Ningbo Hospital of Zhejiang University. The patients/participants provided their written informed consent to participate in this study.

## Author contributions

TX: conceptualization, methodology, formal analysis, and writing-original draft. JY: project administration. QH: data curation. YW, YX, and AW: resources. XG, YH, and YX: funding acquisition. MP and LL: validation and writing-review and editing. All authors contributed to the article and approved the submitted version.

## Funding

This study was supported by Ningbo Health Branding Subject Fund (PPXK2018-04) and Medicine and Health Science and Technology Projects of Zhejiang Province (2022KY305, 2022KY322, 2019KY162).

## Conflict of Interest

The authors declare that the research was conducted in the absence of any commercial or financial relationships that could be construed as a potential conflict of interest.

## Publisher's note

All claims expressed in this article are solely those of the authors and do not necessarily represent those of their affiliated organizations, or those of the publisher, the editors and the reviewers. Any product that may be evaluated in this article, or claim that may be made by its manufacturer, is not guaranteed or endorsed by the publisher.

## Supplementary material

Supplementary Figure 1 Illustrative cases for high NWU with sICH vs. low NWU without sICH. Case 1 **(A–C)**, **(A)** lesion map, green region represents ischemic penumbra and red represents ischemic core. (B) NCCT with hand-drawn ROI, NWU-core = 4.8%, NWU-penumbra = −0.5%. **(C)** Follow-up CT didn't indicate ICH. Case 2 **(D–F)**, **(D)** lesion map, green region represents ischemic penumbra and red represents ischemic core. **(E)** NCCT with hand-drawn ROI, NWU-core = 11.3%, NWU-penumbra = 5.1%. **(F)** Follow-up CT indicates parenchymatous hematoma in the basal ganglia.

Supplementary Table 1 Patient's demographics of group MT vs. non-MT.

Supplementary Table 2 Univariate logistic regression analysis for risks of NWU-core and NWU-penumbra on different types of HT.
