# Supplementary figures and images for: Net water uptake, a neuroimaging marker of early brain edema, as a predictor of symptomatic intracranial hemorrhage after acute ischemic stroke
Source: Front Neurol. 2022 Jul 27;13:903263. doi: 10.3389/fneur.2022.903263 (PMC9363701; doi:10.3389/fneur.2022.903263)

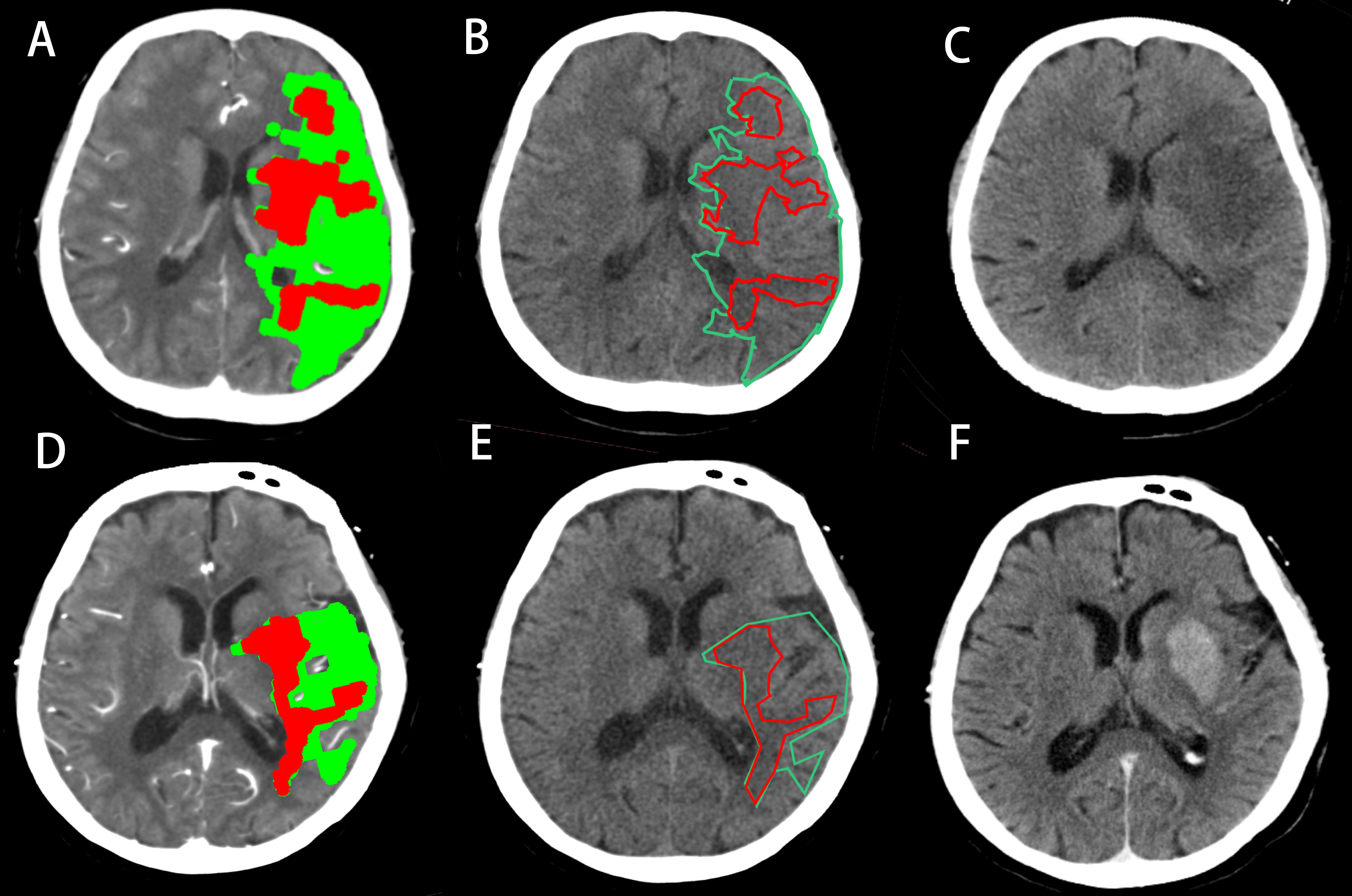

Supplement: Supplementary Figure 1 — Illustrative cases for high NWU with sICH vs. low NWU without sICH. Case 1 (A–C), (A) lesion map, green region represents ischemic penumbra and red represents ischemic core. (B) NCCT with hand-drawn ROI, NWU-core = 4.8%, NWU-penumbra = −0.5%. (C) Follow-up CT didn't indicate ICH. Case 2 (D–F), (D) lesion map, green region represents ischemic penumbra and red represents ischemic core. (E) NCCT with hand-drawn ROI, NWU-core = 11.3%, NWU-penumbra = 5.1%. (F) Follow-up CT indicates parenchymatous hematoma in the basal ganglia. [file Image_1.JPEG]
