# Supplementary material for: Net water uptake, a neuroimaging marker of early brain edema, as a predictor of symptomatic intracranial hemorrhage after acute ischemic stroke
Source: Front Neurol. 2022 Jul 27;13:903263. doi: 10.3389/fneur.2022.903263 (PMC9363701; doi:10.3389/fneur.2022.903263)
Supplement: Supplementary Table 1 — Patient's demographics of group MT vs. non-MT. [file Table_1.doc]

**Supplementary table 1. Patient’s Demographics of Group MT versus non-MT**

|  | **With MT**  **(n=93)** | **Without MT**  **(n=61)** | ***P* value** |
| --- | --- | --- | --- |
| ***Demographics*** |  |  |  |
| Age, mean ± SD | 68 ±13 | 72 ±12 | **0.030** |
| Sex, male (%) | 48 (51.6) | 39 (63.9) | 0.139 |
| ***Comorbidities*** |  |  |  |
| Hypertension, n (%) | 52 (55.9) | 43 (70.5) | 0.090 |
| Diabetes mellitus, n (%) | 12 (12.9) | 5 (8.2) | 0.438 |
| Atrial fibrillation, n (%) | 60 (64.5) | 34 (55.7) | 0.313 |
| Coronary heart disease, n (%) | 12 (13.0) | 7 (11.8) | 0.809 |
| ***Stroke etiology*** |  |  | 0.806 |
| Atherothrombotic, n (%) | 17 (18.3) | 14 (23.0) |  |
| Cardioembolic, n (%) | 56 (60.2) | 34 (55.7) |  |
| Others, n (%) | 20 (21.5) | 13 (21.3) |  |
| ***NIHSS score, median (IQR)*** | 17 (13.5-21) | 17 (8-23) | 0.393 |
| ***Time from symptom onset to CT, min, median (IQR)*** | 288 (200-388) | 253 (192-341) | 0.281 |
| ***Imaging biomarkers*** |  |  |  |
| Ischemic core volume, ml, median (IQR) | 32 (12-48) | 21.7 (4-57.9) | 0.346 |
| Ischemic penumbra volume, ml, median (IQR) | 91 (60-113) | 41.4 (17-80.8) | **< 0.001** |
| NWU-core, %, median (IQR) | 6.4 (3.2-9.6) | 6.0 (2.2-8.5) | 0.302 |
| NWU-penumbra, %, median (IQR) | 2.0 (-0.4-4.0) | 1.5 (-2.2-4.3) | 0.454 |
| ***Hemorrhage transformation (HT)*** | 41 (44.1) | 17 (27.9) | **0.061** |
| HI 1 | 6 (6.5) | 2 (3.3) |  |
| HI 2 | 14 (15.1) | 10 (16.4) |  |
| PH 1 | 8 (8.6) | 4 (6.6) |  |
| PH 2 | 13 (14.0) | 1 (1.6) |  |
| ***Symptomatic intracranial hemorrhage (sICH)*** | 23 (24.7) | 5 (8.2) | **0.010** |

Abbreviation: MT = mechanical thrombectomy, NIHSS = National Institutes of Health Stroke Scale, NWU-core = net water uptake within ischemic core, NWU-penumbra = net water uptake within ischemic penumbra. HT = hemorrhagic transformation, HI = hemorrhagic infarction, PH = parenchymatous hematoma, sICH = symptomatic intracranial hemorrhage.

For continuous variables that follow a normal distribution, data were expressed as mean ± SD, the *t* test was used for group comparison. For non-normal continuous variables, data were presented in the form of the medians (interquartile ranges), and the Mann-Whitney *U* test was applied. Categorical variables were expressed as frequencies (percentages), using the *χ2* test or Fisher's test as appropriate.
