# Supplementary material for: Net water uptake, a neuroimaging marker of early brain edema, as a predictor of symptomatic intracranial hemorrhage after acute ischemic stroke
Source: Front Neurol. 2022 Jul 27;13:903263. doi: 10.3389/fneur.2022.903263 (PMC9363701; doi:10.3389/fneur.2022.903263)
Supplement: Supplementary Table 2 — Univariate logistic regression analysis for risks of NWU-core and NWU-penumbra on different types of HT. [file Table_2.doc]

**Supplementary table 2. Univariate Logistic Regression Analysis for Risks of NWU-core and NWU-penumbra on different types of HT**

|  | NWU-core | | NWU-penumbra | |
| --- | --- | --- | --- | --- |
|  | OR (95% CI) | *P* value | OR (95% CI) | *P* value |
| HT | 1.038 (0.967-1.115) | 0.302 | 1.064 (0.984-1.152) | 0.120 |
| HI1 | 0.972 (0.817-1.156) | 0.749 | 1.069 (0.891-1.282) | 0.474 |
| HI2 | 0.935 (0.836-1.044) | 0.232 | 0.957 (0.863-1.060) | 0.398 |
| PH1 | 1.132 (0.992-1.292) | 0.065 | 1.215 (1.017-1.451) | **0.032** |
| PH2 | 1.164 (1.029-1.316) | **0.015** | 1.285 (1.060-1.556) | **0.010** |
| sICH | 1.114 (1.020-1.217) | **0.016** | 1.268 (1.105-1.456) | **0.001** |

Abbreviation: NWU-core = net water uptake within ischemic core, NWU-penumbra = net water uptake within ischemic penumbra. HT = hemorrhagic transformation, HI = hemorrhagic infarction, PH = parenchymatous hematoma, sICH = symptomatic intracranial hemorrhage.
